# Introducing the NEMO-Lowlands iconic gesture dataset, collected through a gameful human–robot interaction

**DOI:** 10.3758/s13428-020-01487-0

**Published:** 2020-10-19

**Authors:** Jan de Wit, Emiel Krahmer, Paul Vogt

**Affiliations:** 1 Department of Communication and Cognition, Tilburg Center for Cognition and Communication, Tilburg University, PO Box 90153, 5000LE Tilburg, Netherlands; 2 Department of Cognitive Science and Artificial Intelligence, Tilburg University, PO Box 90153, 5000LE Tilburg, Netherlands

**Keywords:** Gesture dataset, Semi-structured elicitation, Human–robot interaction

## Abstract

This paper describes a novel dataset of iconic gestures, together with a publicly available robot-based elicitation method to record these gestures, which consists of playing a game of charades with a humanoid robot. The game was deployed at a science museum (NEMO) and a large popular music festival (Lowlands) in the Netherlands. This resulted in recordings of 428 participants, both adults and children, performing 3715 silent iconic gestures for 35 different objects in a naturalistic setting. Our dataset adds to existing collections of iconic gesture recordings in two important ways. First, participants were free to choose how they represented the broad concepts using gestures, and they were asked to perform a second attempt if the robot did not recognize their gesture the first time. This provides insight into potential repair strategies that might be used. Second, by making the interactive game available we enable other researchers to collect additional recordings, for different concepts, and in diverse cultures or contexts. This can be done in a consistent manner because a robot is used as a confederate in the elicitation procedure, which ensures that every data collection session plays out in the same way. The current dataset can be used for research into human gesturing behavior, and as input for the gesture recognition and production capabilities of robots and virtual agents.

## Introduction

To support studies into non-verbal behavior, and in order to imbue robots and virtual agents with the ability to communicate with us in a human-like way, there is a need for structured, labeled, and large-scale datasets of human-performed gestures (Argall et al., 2009; Ortega & Özyürek, 2020). Ideally, these datasets contain gestures that are recorded in an ecologically valid way, and stored in a format that lends itself to automated analysis. Furthermore, it should be possible to collect additional data in a consistent manner, for example in order to include gestures for additional concepts or to replicate data collection in a new (demographic or cultural) context. With the aim of collecting such a dataset of iconic gestures in a naturalistic setting, we developed a game of charades with a humanoid robot. This game was used to record a large number of iconic gestures from a diverse group of participants at the NEMO science museum and at the Lowlands Science event, as part of the Lowlands music festival. Both events took place in the Netherlands.

The resulting dataset of motion-capture recordings for 35 different objects, such as animals and musical instruments, has a number of unique aspects that make it a valuable tool for studies and applications involving iconic gestures. First, it is a large-scale set both in terms of the number of unique recordings, as well as the number of participants that are included. Second, the participants were free to choose how they wanted to portray the concepts using silent gesture. Third, a broad range of demographic backgrounds—children and adults, several different cultures—is represented in the dataset. Fourth, to our knowledge, no existing research has looked into the degree to which people tend to change their gesturing approach when an interlocutor fails to recognize their first attempt at depicting a concept. The current dataset provides support for first explorations into these repair strategies, and how often they were used. The combination of these four aspects has allowed us to capture different variations that are likely to occur in gesture production. This enables researchers to answer various research questions related to human-performed gestures, and factors that could potentially influence gesturing behavior.

The dataset contains two-dimensional and three-dimensional motion-capture recordings of the participants performing the gestures. These are stored in a consistent format, which makes the set suitable for automated, large-scale gesture analysis, as well as various applications in the field of artificial intelligence such as gesture production and recognition by virtual agents and robots. Automatic gesture recognition is often done only for well-defined gestures, where the system knows what motion to expect. However, this means that people are limited in choosing their preferred way of depicting a concept using gestures. The current dataset allows researchers to explore whether it is possible to create recognition systems that can handle a variety of different representations for the same concept. An agent’s gesture production capabilities can also be based on the recordings in our dataset, thus supporting studies into the added value of using data-driven gestures, and how comprehensible these are compared to manually designed gestures. Because the game of charades is made publicly available, it is possible to extend the dataset to include new concepts, or to record additional gestures in different cultures or contexts.

### Gesture and interaction

Manual gestures (Kendon, 2004) are an integral part of our communicative abilities: they help guide the recipients’ attention, and support the comprehension of information that is being conveyed in speech (Goldin-Meadow, 2005; Hostetter, 2011). They serve a purpose for the person producing the gestures as well, by helping them to be more fluent and rich in their speech (Cravotta et al., 2019; Hostetter, 2011). In this work, we focus on iconic gestures, a specific subset that includes movements where the depicted shape is related to the concept that is referred to (McNeill, 1992). For example, an iconic gesture for the concept of a *bird* could consist of gracefully moving one’s hands up and down repeatedly, as a reference to the act of flying. Iconic gestures in particular play an important role in supporting speech comprehension (Kelly et al., 1999), especially in noisy environments (Drijvers & Özyürek, 2017). Furthermore, people with certain impairments that prevent them from (fully) using or understanding speech, such as aphasia (language impairment due to brain injury), can benefit from gestures as a communicative and therapeutic device (van Nispen et al., 2018). Finally, research in the field of education has shown that iconic gestures can be used as a means of providing scaffolding to support the learning process (Alibali & Nathan, 2007). In light of this important role of iconic gestures in communication and education, with the current work we aim to provide a dataset and recording method to support further studies into the intricacies of gesturing behavior.

Because gestures are a natural and intuitive way for us to communicate with each other, researchers have started to explore whether we can use them to interact with machines as well (Karam & Schraefel, 2005). Recent technological developments enable everyday computer systems to track body posture and hand gestures in a minimally invasive fashion, which allows for the use of gestures as input device instead of using traditional controllers such as a mouse and keyboard (Lun & Zhao, 2015). This is especially relevant when these interactions involve artificial agents, either virtual or robotic, with whom we expect to be able to communicate by means of natural language (Bartneck & Forlizzi, 2004). Ideally, these agents should be able to understand the gestures produced by humans, as well as produce gestures of their own to support their social and communicative behaviors (Fong et al., 2003). We recently investigated whether gestures are able to support a robot’s teaching efforts and found that children of 4–6 years old were more engaged with the interaction and showed higher learning gains when they interacted with a robot tutor that performed iconic gestures while teaching second language vocabulary, compared to one that did not use gestures (de Wit et al., 2018).

There are various methods—or modes of representation (Müller, 2014)—to describe a certain concept by means of iconic gestures. For example, one could gesture by outlining the physical shape of an object, such as the handle and bristle of a toothbrush, or by performing the act of using or interacting with the object: brushing our teeth. Although many concepts appear to have a default mode of representation (Dargue & Sweller, 2018; Masson-Carro et al. 2017; van Nispen et al. 2014, 2017; Ortega & Özyürek 2016, 2020), this is known to vary based on aspects such as the cultural background (Kita, 2009) or age of the performer (Jain et al., 2016; Masson-Carro et al., 2015; Sekine et al., 2018; Stites & Özçalışkan, 2017). The study by Sekine et al., (2018) showed that 3-year-old children had a tendency towards using their entire body to represent the protagonist when retelling a story (character viewpoint), and they used a larger gesture space compared to adults. The adult participants instead performed gestures from the perspective of an outsider looking in (observer viewpoint), representing and manipulating the protagonist as a smaller, imaginative object. Even when performing the same gesture, Jain et al., (2016) observed that children of 5–9 years old tend to produce faster and less coordinated motions than adults.

These variations in the way we depict concepts using gestures poses two challenges when attempting to imbue robots with the ability to understand and produce these motions. First, the robot-performed gestures are often designed by researchers using common animation techniques such as key framing. These researchers may not necessarily belong to the same demographic as the people that will end up interacting with the robot, and the robot’s gestures might therefore fail to match the recipient’s preferred modes of representation (Ortega and Özyürek, 2020), which could cause miscommunication. Second, a robot with social intelligence should also be able to recognize gestures performed by others, which are likely to include a number of variations for the same concept. Therefore, both the production and recognition of gestures by a robot would benefit from a data-driven approach, where many examples of people performing gestures are used to inform the robot’s gesture production and recognition capabilities. There is a call for more data in the field of gesture studies as well (Ortega & Özyürek, 2020), in order to investigate whether patterns that we see on a smaller scale, e.g., regarding default modes of representation, can be generalized to a broader range of concepts or demographics. This ongoing research into human-performed gestures can be further supported by tools that have recently been developed to support automatic extraction of features such as size, velocity, and sub movements from three-dimensional gesture recordings (Pouw & Dixon, 2020; Trujillo et al., 2019), which enable analysis of gestures on a large scale. In order to improve the design of robot-performed gestures, and to support further studies into gesturing behavior, we have set out to collect such a dataset of three-dimensional recordings of human-performed gestures in a naturalistic setting.

These datasets can be collected in a number of different ways. For example, in recent work in the field of human–robot interaction, gestures were automatically extracted from natural interactions, such as recordings of TED talks (e.g., Ghosh et al. 2019; Hua et al. 2019; Shimazu et al. 2018; Yoon et al. 2019). These recordings were never intended to be used for this purpose, which means that the gestures that occur are naturalistic, but there is also no control over which (types of) gestures are performed. As a result, these gestures can be used for generating human-like co-speech gestures, but are less suitable for studying iconic gestures. The present work therefore focuses on the use of an elicitation procedure, which involves recording a number of participants as they perform gestures belonging to a predefined set of concepts. These concepts are presented to them one by one, either verbally or using visual cues. This method has also been used in the field of human–computer interaction, initially for the design of gesture interactions with a touch surface (Wobbrock et al., 2009), and subsequently for full-body gestures (e.g., Silpasuwanchai & Ren 2014), also with children (Connell et al., 2013). The goal in the context of human–computer interaction is to reach consensus on the gesture that best describes a particular action within a computer system (Vatavu, 2019), such as shooting and reloading a gun in a videogame. Elicitation studies enable the collection of gesture datasets in a structured manner. It is possible to ask participants to perform examples of concrete motions (e.g., “claw like a bear”), but a more diverse set with different modes of representation can be collected by giving participants more general cues (e.g., “bear”). However, the data resulting from elicitation studies can be relatively unnaturalistic because participants are prompted to perform these gestures, often in a controlled setting, and they are aware of the goal and context of the study.

In order to obtain more naturalistic results, Eisenbeiss (2010) suggests the use of a semi-structured elicitation procedure, where the context is kept as natural as possible by having participants engage in a “game”, while still providing prompts to elicit certain responses. One example of a gameful approach is the director-matcher task. In this task a participant is assigned the role of director and is asked to describe a complex abstract shape to another participant, the matcher, who has to recreate this shape without having seen it (Krauss and Weinheimer, 1964). In gesture research, this method can be used to elicit a combination of speech and spontaneous gestures (e.g., Holler & Wilkin 2011). This task can be considered an unstructured elicitation procedure, with little control over which exact gestures will be produced. Semi-structured and game-like approaches appear to be understudied in research. One example is Bartertown (van den Heuvel, 2015a), where participants engaged in a science-fiction game in which they were asked to communicate the appearance of certain primitive shapes to a virtual agent by means of gesturing. The recorded gestures were then mirrored by the virtual character and the participant was asked to confirm whether they were recorded correctly, and to re-do them if needed. Later in the game, other virtual characters performed gestures that were previously recorded from different participants and the current participant was asked to label these, essentially covering both the generation and labelling of data in one sitting.

To our knowledge, the potential use of repair strategies when there is a breakdown in non-verbal communication, both between two humans and between a human and a robot, has not yet been studied. However, we can find inspiration in the field of human–computer interaction, where mid-air (Walter et al., 2013) or touch gestures (Bragdon et al., 2009; Bragdon et al., 2010) can be used to trigger certain software commands. In this case, it takes time and multiple attempts for the user to explore which gestures are available, and to learn how they should be performed in order to trigger the correct functionality. Bragdon et al., (2009) found that a number of participants in their study either did not discover some of the available touch gestures at all, or they were unable to perform them in the proper way to trigger the functionality of the interface. This indicates a mismatch between the designer’s expectations of the gestures that people will perform when interacting with their software, and the gestures that users actually come up with and the strategies they use to explore the space of potential gestures. We can apply the same principle to our studies in human–robot interaction: If we design the robot’s gesture production and recognition capabilities solely on our own frame of reference, we are bound to introduce a certain degree of miscommunication. Therefore, it would be better to start by observing interactions, and then inferring common gesturing and repair strategies from these observations. Miscommunication can also occur when technology such as automatic speech recognition or, in our case, gesture recognition is not successful at recognizing the user’s input correctly, a situation in which users can rely on multiple modalities for correcting these recognition errors (Suhm et al., 2001).

### Existing gesture datasets

Several gesture datasets have been presented in literature, with various goals ranging from studies into human gesturing behavior, to applications related to artificial intelligence such as gesture recognition and gesture synthesis for virtual agents (e.g., Ortega & Özyürek 2020; Sadeghipour et al. 2012; Vatavu 2019). These sets differ in scale, in terms of the number of concepts included and the number of people recorded. Furthermore, different sensors were used to record the gestures, including traditional video cameras, depth sensors such as the Microsoft Kinect, and tracking devices that were held by or attached to the participants performing the gestures. These existing datasets can further be categorized by the elicitation procedure that was used, either (semi-)structured with specific cues, or unstructured where all of the gestures that were produced spontaneously during a broad task were recorded. An example of the latter approach is EGGNOG (Wang et al., 2017), where participants were given a collaborative task to recreate a structure out of wooden blocks from a picture. This resulted in a total of 8 h, collected over 360 trials with 40 participants, of naturally occurring gestures along with speech (for a subset of the trials). Another example is SaGA (Lücking et al., 2010), in which 25 pairs of participants were asked to perform tasks that involved giving directions and describing various scenes containing multiple objects. The resulting set contains recordings of speech and non-verbal behavior from 25 dialogues, including a total of almost 5000 iconic and pointing gestures.

A literature review by Ruffieux et al., (2014) describes 15 datasets that were compiled specifically for developing and evaluating gesture recognition algorithms, which were collected using a structured elicitation procedure in a controlled setting. In most of the work discussed in this survey, the gestures do not refer to real-life objects, instead they are motions that were designed specifically to trigger certain actions during human–computer interactions (e.g., swiping to the right in the air to trigger the next song to play). Furthermore, participants were often given concrete prompts that already steered towards a particular aspect of the target concept, thus already implying a desired mode of representation, such as the aforementioned “claw like a bear” instead of just “bear”. Only in the 3DIG dataset (Sadeghipour et al., 2012) participants were given the freedom to choose which representation technique (e.g., shape versus action) to use. This transforms the challenge of gesture recognition into being able to recognize any gesture that represents an object, rather than one specific motion. This form of gesture recognition is more realistic when communicating with (virtual) agents, where the focus lies on being able to understand which object is being described, regardless of individual differences in preferred gesturing strategy. In addition to their role in gesture recognition, such extensive and varied datasets can also be used for research into gesturing behavior in general. The 3DIG set contains recordings from a total of 29 participants, who were presented with ten primitive objects and ten complex objects such as *house* or *apple*. The aforementioned semi-structured elicitation procedure Bartertown (van den Heuvel, 2015a) also resulted in a publicly available dataset (van den Heuvel, 2015b), which includes three-dimensional gesture recordings of 36 participants each depicting four shapes, with eight different shapes in total included. A recent example from the field of gesture research is the work by Ortega and Özyürek (2020), where 20 participants were asked to provide silent gestures for 272 different concepts across five semantic domains (manipulable and nonmanipulable objects, actions with and without objects, and animate entities), and were also given the freedom to choose their gesturing strategy.

Although the previously discussed datasets were all recorded with adult participants, there are datasets that include gestures performed by children as well. Vatavu (2019) published a set containing 1312 whole-body gestures in total across 15 different concepts including objects such as flowers as well as actions such as climbing a ladder or turning around. These gestures were recorded from 30 children between the age of three and six. Children in this case were given concrete instructions on how to represent the concepts, for example to “Draw a flower in mid-air”. The Kinder-Gator dataset (Aloba et al., 2018) contains recordings of 58 different gestures related to the categories warm-up, exercise, mime, and communication, such as “Motion someone to come here”. These were recorded from ten children (aged five to nine) and ten adults.

Our survey of related work identifies several gaps in the datasets that are currently available. In most cases, participants were given concrete prompts for the types of gestures to perform, which makes these datasets unsuitable for studying individual differences in modes of representation. In addition, literature has found that gesturing strategies tend to differ between children and adults, however the only set from our survey of related work that includes both children and adults performing the same gestures is Kinder-Gator (Aloba et al., 2018). A limitation of this elicitation study is that the number of participants is relatively small (ten children and ten adults), they were given concrete prompts, and only few of the concepts elicited iconic gestures where the motion was semantically related to the concept being depicted. As a result, while this does support studies into quantifiable differences in motion characteristics (e.g., speed, size) between adults and children, it does not provide the variation needed to investigate differences in modes of representation. Finally, to our knowledge there is no iconic gesture dataset that includes the same participant performing a second gesture for the same concept, after they realize that the first example is not understood by the confederate. These second attempts would give insight into repair strategies that people tend to use when miscommunication occurs.

In our review of related datasets, we also found that generally none of the materials from the elicitation procedure that were used to collect the data are made available. This impedes potential future extensions of the datasets. In addition, the elicitation procedure relies on a human confederate, who has to follow a specific protocol. By having a robot perform this procedure instead, it is possible to replicate the data collection process in a consistent manner. In the present study, we aim to address the limitations of currently available iconic gesture datasets in two different ways: 1) by publishing a dataset that includes recordings from children and adults, who were free to choose their preferred mode of representation, and who were asked to perform a second gesture in case miscommunication occurred; 2) by making the game of charades publicly available, thereby allowing other researchers to further extend the dataset with different concepts, or in different cultures and contexts. Our dataset includes three-dimensional motion capture recordings from a depth camera, and two-dimensional motion capture data that were extracted from video recordings post hoc using an algorithm. Both formats have certain advantages and drawbacks, which will be discussed later in the paper.

The next sections describe the game of charades with a robot that was used as elicitation procedure, followed by details regarding the technical implementation, and a description of the resulting dataset.

## Gesture elicitation procedure

The game of charades was set up at the NEMO science museum in Amsterdam for 2 weeks in July and August, 2018, and at all 3 days of the Lowlands music festival, which took place August 17–19, 2018. Visitors to the science museum and music festival were free to observe the study and, if they were at least 5 years old, could choose to volunteer as a participant. The study was carried out with approval from the research ethics committee of the Tilburg School of Humanities and Digital Sciences at Tilburg University. Participants, or their legal guardian in case they were younger than 16 years old, had to sign an informed consent form in order to participate, with which they also agreed that their data could be incorporated into the dataset. We also obtained verbal assent of all participants, and asked whether or not their interactions could be recorded on video in order to be able to extract two-dimensional motion capture data. These video recordings were optional, while the motion capture recordings from the depth sensor were required in order to participate.

### Participants

A total number of 317 visitors to the science museum participated in the study, and 116 at the music festival. Due to children not finishing the game, or participants that took part in a demonstration of the system without wanting to have their data stored, we had to exclude five participants from the science museum. The total number of participants whose data were included, as well as their demographic information, is displayed in Table 1.

**Table 1:** Participant information

	NEMO	Lowlands	Total
Participants	312	116	428
Gender	157 male	49 male	206 male
	149 female	67 female	216 female
	6 unknown		6 unknown
Age (Y;M)	12;11	28;4	17;2
	*SD* = 10;7	*SD* = 8;8	*SD* = 12;2
	11 unknown	2 unknown	13 unknown
Countries	27	4	28
		1 unknown	1 unknown

### Materials

**Fig. 1:**
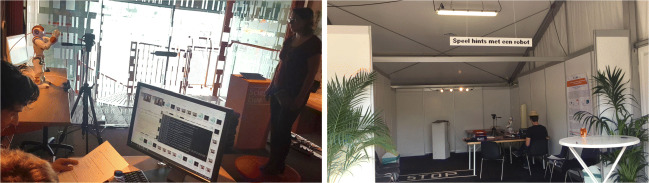
Photographs of the set-up at the NEMO science museum (*left*) and the Lowlands music festival (*right*)

The experimental set-ups at the science museum and the music festival are shown in Fig. 1. The system that was used in the experiment included a SoftBank Robotics NAO V5 robot, a Kinect V2 for recording, a Microsoft Surface tablet as the interface for the participant and a control panel running on a separate laptop or computer for the experimenter. A Logitech C920 webcam was also included to capture video from which two-dimensional pose data were extracted after data collection was completed.

**Fig. 2:**
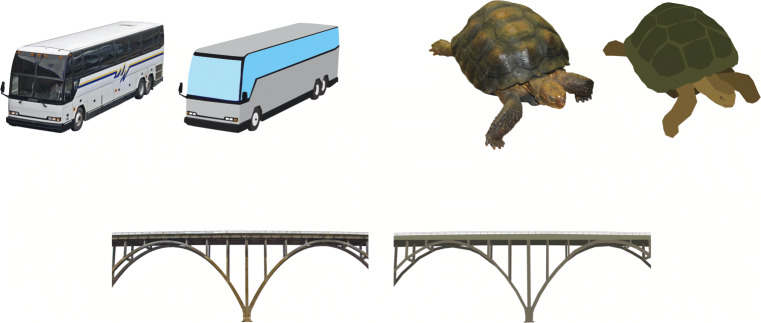
Three examples (*bus*, *tortoise*, and *bridge*) of photographs from the BOSS set and the corresponding traced images that were used in the game of charades—*tortoise* was also renamed as *turtle*

Thirty-five different concepts were included in the experiment for participants and the robot to depict. These were picked from the Bank of Standardized Stimuli (BOSS) containing photographs of a multitude of objects (Brodeur et al., 2014). Because we expected a substantial part of our participants to be younger children, we traced and colored the photographs to make them look more cartoon-like (Fig. 2). The age of acquisition (Kuperman et al., 2012) was used as a guideline when choosing the concepts to ensure that the youngest participants (5 years old) would be familiar with them. The concepts were divided into five different categories, with seven concepts in each category: animals, static objects, tools, musical instruments, and means of transportation. These categories were chosen in order to capture a diverse range of concepts, including both animate and inanimate objects, objects of varying sizes, and objects that afford different types of interactions (e.g., walking on a bridge, handling a toothbrush). To get a realistic idea of the robot’s gesture recognition performance, several of the concepts were chosen to be similar to each other in terms of the default gesture we expected participants to use, such as car and bus, or xylophone and drum set. Appendix A contains an overview of all the included concepts.

### Procedure

After visitors showed an interest in participating in the study, they were presented with a letter containing general information about the goals of the study, an explanation of the interaction with the robot (i.e., that they would play a game involving gestures), the nature of the recorded data (with a picture illustrating the output of the Kinect sensor), and details on the way their data would be collected and managed. To get an overview of what the game was like, visitors were also free to observe participants that were currently playing. After signing the informed consent form, their participant number was entered into the control panel. If the participant allowed their video to be recorded, a checkmark was set which enabled the system’s video recording functionality. Additionally, participants could receive a link to a website with their own motion capture recordings. If they were interested in receiving this link, their e-mail address was entered into the control panel. The game was then started by the researcher by pressing a button on the control panel. The robot stood up and started “breathing” (shifting its weight from one leg to the other and swaying its arms slowly—a built-in feature of the NAO robot) to make it look more active and alive. It also blinked its eyes every five seconds by turning the LEDs off and on again. A language choice between Dutch and English was shown on the tablet, which affected the robot’s speech as well as the labels for the items presented on the tablet.

**Fig. 3:**
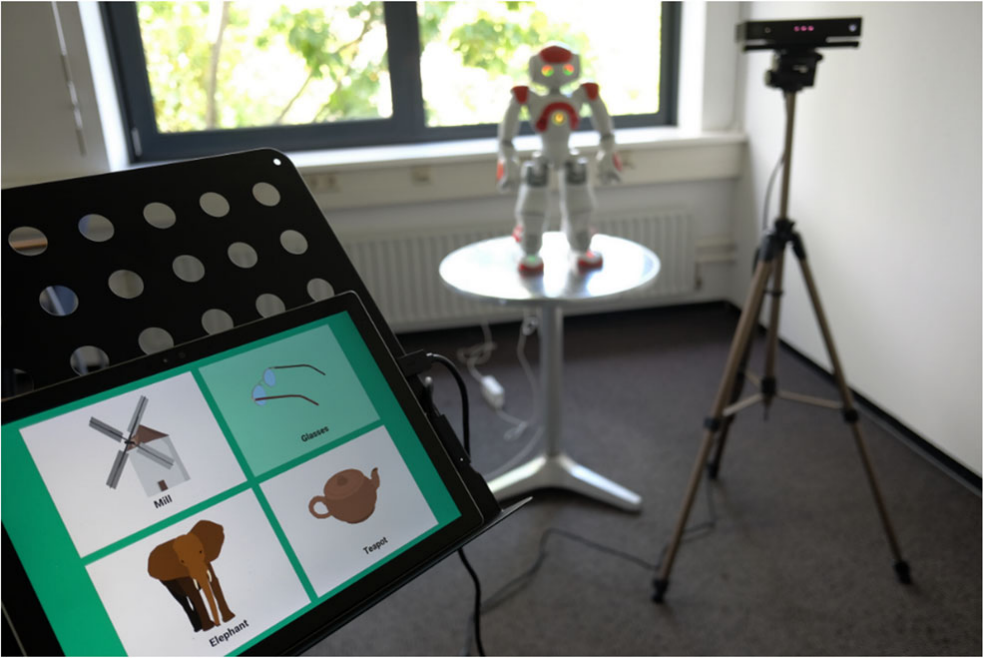
During the practice round, a participant guesses the gesture for *glasses* that the robot had just performed

The participant was invited to stand close to the tablet device so that they could operate it, and in front of the Kinect camera, which was moved approximately to the participant’s shoulder height. The researcher then gave a short introduction to the game, indicating that the robot would only be able to see their upper body motion and instructing the participant to stand still with their hands pointing down at their sides when they were done gesturing. After choosing a language, the robot greeted the participant and explained the basics of the game to them. This was followed by a practice round, where the robot performed a prerecorded gesture to depict *glasses*, and the participant had to guess by selecting the corresponding image out of four different options (Fig. 3).

**Fig. 4:**
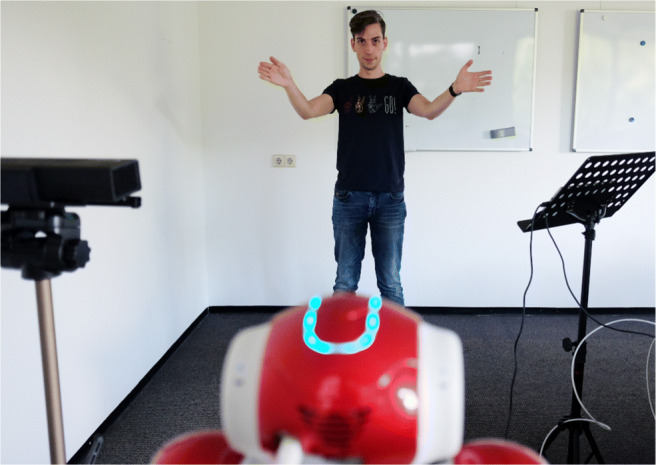
Second part of the practice round: The participant performs a gesture for *ball*

Regardless of whether the participant guessed correctly or incorrectly, the game then proceeded to the second part of the practice round where the participant was asked to show a gesture for the object *ball*. After taking time to think of a way to depict the ball, the participant triggered a countdown by pressing the start button on the tablet, after which he or she could start performing the gesture (Fig. 4). Participants were instructed to stand still after completing a gesture, which enabled the system to automatically detect when to stop recording. In a later version of the system (used at Lowlands), there was also a button for the researchers to manually stop the recording. After the recording was stored, the robot tried to guess the gesture, which for this introductory stage was hard-coded to always be the correct guess regardless of the actual gesture that was performed by the participant.

After guessing the gesture, the robot displayed a top three of candidates for its guess along with a percentage showing how much confidence the robot had in that particular candidate. This step was included to give participants insight into the robot’s thought process and reasoning behind its guesses. As with the other parts of the practice rounds, this was fixed and always showed the same three concepts with the same confidence values. All of the items used in the practice round were not part of the 35 concepts that make up the final dataset.

**Fig. 5:**
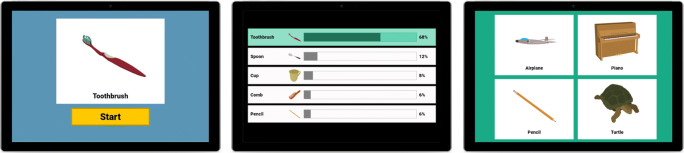
Screenshots of the tablet screen during the game of charades. *Left*: the participant’s turn to perform a gesture for *toothbrush*; *middle*: when guessing, the robot shows its top five candidates, of which it will guess the first one (in this case *toothbrush*), the correct answer is highlighted; *right*: the robot just performed a gesture and the participant has to choose the matching item

The participant then played five turns of the actual game, which were identical to the practice round except now with a selection of ten out of the 35 included concepts—five to be depicted by the robot, and five by the participant. These concepts were chosen randomly, while ensuring that the number of total recordings across participants was equally distributed between the 35 concepts. The robot now based the gestures it performed on recordings from previous participants. In addition, it used a gesture recognition algorithm to try and identify the gestures performed by participants, and showed the actual top five candidates proposed by the algorithm. If the robot or participant guessed incorrectly a second attempt took place for the same concept. In many cases, this meant that the robot chose a different recording to perform for the concept. The four answer options on the tablet did not change, therefore participants had to guess from three items in the second round, because they already knew that one of the four original items was incorrect. The participants were also free to change their gesturing strategy for their second attempt (e.g., come up with a different mode of representation altogether, or repeat their previous gesture but then bigger or slower), although they were not actively asked to do so. The gesture recognition algorithm was purposefully implemented, even though it would mean an unequal number of repair attempts per concept, and per participant. We felt that it was important to offer a transparent and fair game experience to the participants, since we were working in two real-world environments. Furthermore, if participants would realize that the robot’s guessing performance was controlled by us, they might not take the experiment seriously anymore, which would have negatively affected the quality of the recorded gestures. Figure 5 shows the information displayed on the tablet at various stages during the game of charades. The interaction with the robot lasted approximately 10 min.

## Technical implementation

In this section, we present a general overview of the game of charades that was used as a semi-structured elicitation procedure. Additional details are available with the publicly available source code.^1^ The implementation consists of several modules that communicate with each other using a local network connection. A key advantage of this architecture is that modules that have been developed in different programming languages can still work together. The current system contains a combination of C# for Kinect, Javascript for the tablet interaction, and Python to drive the robot. In addition, each module is freely interchangeable as long as it sends the expected output to other modules and is able to handle the provided input. This means that different algorithms such as a better performing gesture recognition approach can easily be added in the future. In a similar vein, it is possible to support other robots or virtual agents as well as other recording devices without having to rebuild the entire system.

The current configuration uses a SoftBank Robotics NAO V5 robot, which is a commercially available and widely used humanoid robot. With 25 degrees of freedom it is more limited than humans in performing gestures. Most notably, it is unable to move its three fingers individually, so it is only able to open and close its hand in a gripping motion. In addition to the robot, the system requires a participant-facing tablet on which the game itself runs, and a computer where data can be stored and from which the researcher can control the experiment. We used a Microsoft Surface tablet for both the participant and the researcher. The human gestures were recorded using a Microsoft Kinect V2 depth camera, a device that was originally designed as an input device for the Xbox 360 gaming console but can be connected to a computer by means of an adapter. This device has since been discontinued but alternatives are available, including an updated version of the Kinect (Azure) which we aim to support with future updates to the source code. The robot and the devices for the participant and researcher were connected to a router via ethernet cables to ensure a stable connection. In the next two paragraphs we will briefly discuss the gesture recognition and production modules, two key components of the system.

### Gesture recognition

To ensure that both the robot and the human participant were playing the game of charades fairly, both parties had to observe a gesture from the other player and then guess which concept it tried to describe. We therefore decided to implement an algorithm for the robot’s gesture recognition capabilities. Because one of the potential use cases for our dataset was to train gesture recognition algorithms, this also enabled us to verify that the dataset was indeed suitable for this task. Finally, we could monitor the robot’s gesture recognition performance as it interacted with people in a real-world setting and added new examples to the dataset.

Motion capture recordings such as the ones obtained from our game of charades are complex time series that describe three-dimensional locations of different joints (e.g., elbows, hands) over time. Therefore, even if two gesture recordings relate to the same concept and the performer used the same strategy to depict this concept, differences in speed, size of the movement, or the number of times a particular motion was repeated make it difficult to identify these similarities between gestures. A commonly used approach to compensate for these differences, particularly in speed, is dynamic time warping (e.g., Arici et al. 2014), which is able to match similar gestures even if they are not synchronized and move at different speeds. However, this method does not differentiate between motions that are crucial parts of the gesture, and the noise that stems from random movement or measurement errors during the recording of the gesture. It is also not robust to differences in participants’ height, distance to the camera, or the size of the gesture, which may cause the joints’ locations between two recordings to be far apart while the overall motion is in fact quite similar.

**Fig. 6:**
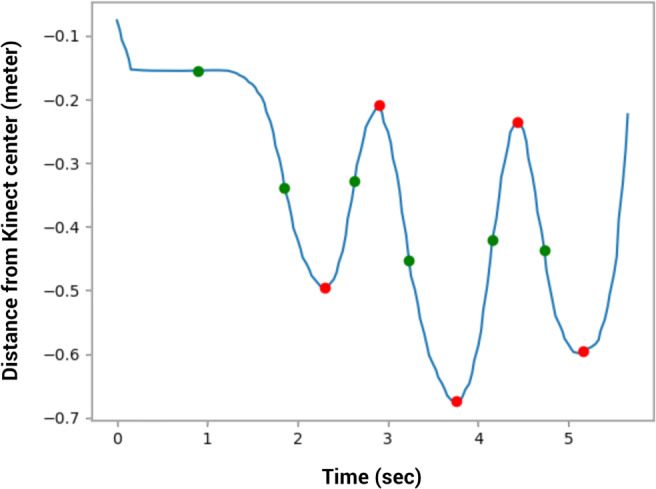
Inflection points (*green*) and peaks (*red*) of a motion trajectory

In order to distinguish between important movements and noise, and to also correct for differences in location due to the position or height of the participant, a pre-processing step is performed to identify salient features of the gestures, also known as *primitives* (Ramey et al., 2012). We based our approach on the work by Cabrera and Wachs (2017) by using the *inflection points* of the hands’ motion trajectories, combined with peaks in the hands’ position (Fig. 6 shows a time series trajectory where inflection points and peaks are marked). Research suggests that inflection points are important features for humans to remember and reproduce gestures (Cabrera et al., 2017). To also take into account differences between participants’ location and height and the size of the gesture, instead of the recorded absolute joint positions we use the positions relative to other joints. For example, we calculate whether the hand was in front of or behind, and above or below the shoulder. Cabrera and Wachs (2017) call the resulting sequence of inflection points and relative locations the *gist* of the gesture. One limitation that remains is that the same gesture could be performed at different positions relative to the body. A gesture for *ball* performed above the shoulders would therefore result in a different description than the same gesture performed in front of the body, below shoulder height.

The former preprocessing steps result in a feature vector describing salient points in the trajectory of the gesture. This feature vector consists of 14 dimensions that include the peaks near inflection points of the motion trajectory of the left hand relative to the left shoulder, the right hand relative to the right shoulder, the left hand relative to the right hand, and the spine at shoulder height relative to bottom of the spine (to measure bending/hunching). These peaks are all extracted from the X, Y, and Z trajectory, resulting in 12 dimensions. Each of these dimensions is a variable length text, which includes the location of the joint relative to the other joint (this is simplified by dividing the physical space into numbered quadrants), and whether at the inflection point the trajectory moved from convex to concave, from concave to convex, or whether it was a stationary point (+, -, or 0). Depending on the duration of the gesture and the number of salient points found within the trajectories of the limbs, one such dimension could contain between 0 and 39 salient points (*M* = 1.95,*SD* = 2.47 points). Each salient point is described by 2 characters of text: a quadrant identifier, and the type of inflection point. The last two dimensions of the feature vector are the percentage of the time the left and right hands were opened.

The next step is to find feature vectors of previously recorded gestures that are similar to that of the newly observed gesture. As a measure of similarity between gestures, we used the Needleman–Wunsch alignment score (Needleman & Wunsch, 1970), applied to the 12 dimensions of the feature vector separately. The similarity matrix is included with the published source code of the system. The difference in percentage of time that the hands were open was then subtracted from the similarity score. This helped the algorithm to distinguish between gestures that look similar if the hands are not taken into consideration, such as pretending to play the piano (open hands) and xylophone (closed hands). After calculating the alignment score between the new gesture and all existing ones in the set, the k-nearest neighbors algorithm (Altman, 1992) was used to determine to which concept the gesture was most likely to belong. This is done by taking the *k* gestures with the highest alignment scores, in other words the *k* recordings in the set that are most similar to the gesture we are trying to recognize. The value of *k* was set to $\sqrt {N}/2$, where *N* is the number of total recordings in the dataset. However, the maximum value of *k* was set to 8 to ensure that the algorithm remained computationally feasible. This was determined empirically while developing the system, so it is possible that this is not yet the optimal value for *k*.

From the neighbors, the concept that occurred most often was chosen as the robot’s guess (majority voting). For example, if the eight closest matches included four recordings belonging to spoon, three to comb and one to toothbrush then the new gesture would be classified as spoon, and this is what the robot would then guess. If two concepts were tied (e.g., both four matches), the neighbor with the lowest similarity score was removed from the set of neighbors, and this process was repeated until there was one concept that had the largest number of matching neighbors.

**Fig. 7:**
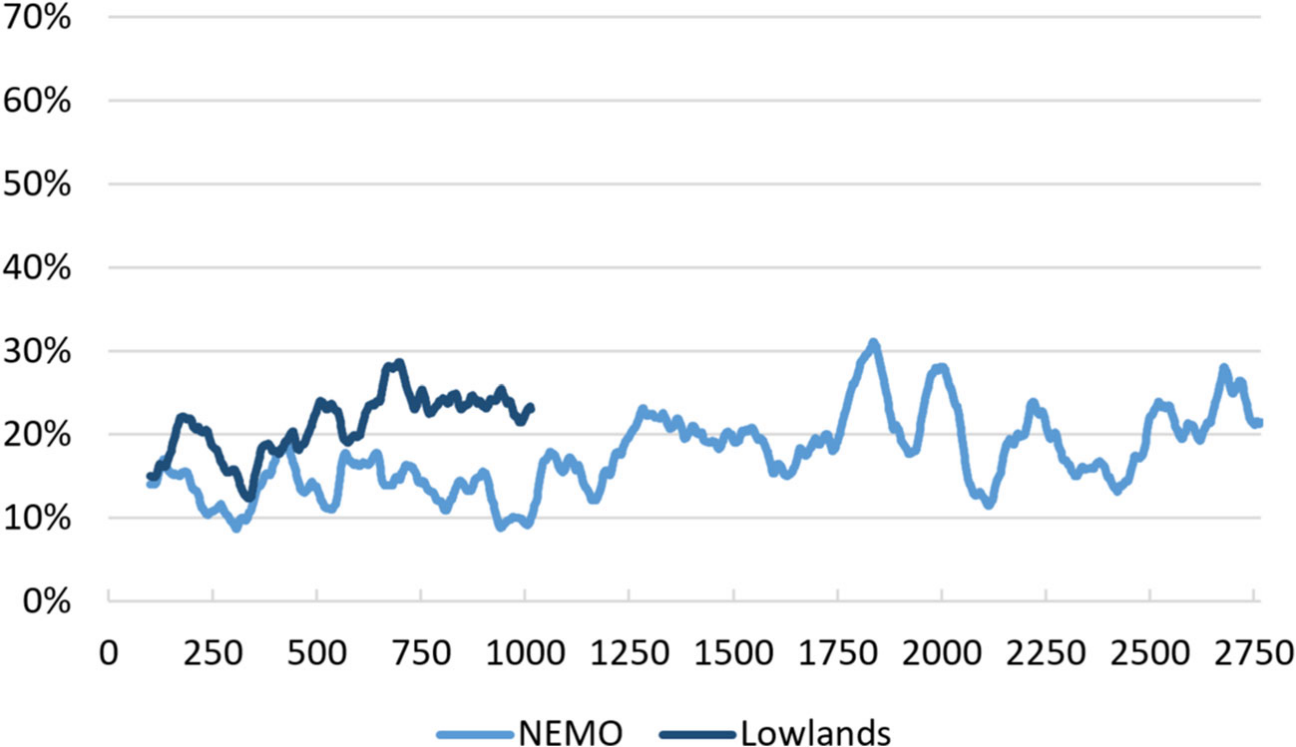
Moving average of the percentage of correct guesses by the robot

All of the robot’s guesses were logged while the system was deployed at the science museum and the music festival in order to get an overview of the gesture recognition performance and how this developed as more data were added. For both events, we initialized the dataset with three recordings for each of the 35 concepts, performed by one of the researchers. This was the starting point to which the system automatically started adding new recordings. Figure 7 shows the moving average, with an interval of 100 recognition attempts and exponential smoothing (*α* = .1), of the robot’s gesture recognition performance over time as it gained more data. Participants who did not want their data included in the analyses have been excluded. The average recognition rate was 17.7% at the NEMO science museum, and 21.0% at the Lowlands festival. Chance level is approximately 2.9%—1/35 for first attempts, and 1/34 for second attempts at guessing. The Lowlands set contains less data because the system only ran for 3 days at that location, compared to 14 days at NEMO.

### Gesture production

The recorded gestures do not contain any visual information, essentially turning the performer into a stick figure. This results in a loss of information compared to regular video recordings: context and facial expressions are missing, and subtle motions may not have been picked up by the Kinect camera. A further loss of information occurs when trying to automatically translate these recordings to a robot with fewer degrees of freedom, less smoothness in its motion, and a smaller reach than a human. However, if this automatic translation were to work while preserving the comprehensibility of the gestures, the robot would have the possibility to imitate human-performed gestures, so that the gestures no longer have to be designed by hand.

To measure the comprehensibility of the gesture recordings and the impact of the loss of information resulting from the recording and translation steps, we had the robot directly use gestures that were previously recorded from other participants. We used an existing implementation to translate the joint locations as they were recorded by Kinect into the yaw, pitch, and roll values needed by the robot (Suay & Chernova, 2011). Because it is not possible for the robot to perform certain motions as fast as a human can, the recordings were slowed down and then sampled at 300ms intervals. In addition, there were recordings where the system did not register that the gesture had ended, and thus also captured noise at the end. Therefore, only a maximum of ten seconds of the recordings were performed by the robot.

Each recording had a *weight* assigned to it, which started at 0 and was updated after the robot had performed this particular recording to a participant. If the participant guessed the corresponding concept correctly, the system increased the weight of this gesture. If the participant chose an incorrect answer, the system decreased the weight. These weights were then used when deciding which recording to use next. To make it easier for the participant to guess a gesture correctly, the robot could perform the recording with the highest assigned weight (the one that had been guessed correctly most often in the past). On the other hand, the robot could also avoid the highest scoring example and explore alternatives instead. To get diverse ratings while still providing participants with a good chance to win, in the current set-up we implemented a 60% chance that the “best” example would be used (exploitation), and a 40% chance that any other recording would be performed by the robot (exploration). Although it would have been possible to ensure that each gesture would receive an equal number of ratings, we opted for this exploration-exploitation approach to lower the difficulty for participants to win the game, and to automatically filter out incorrect or unclear gestures (noise).

**Fig. 8:**
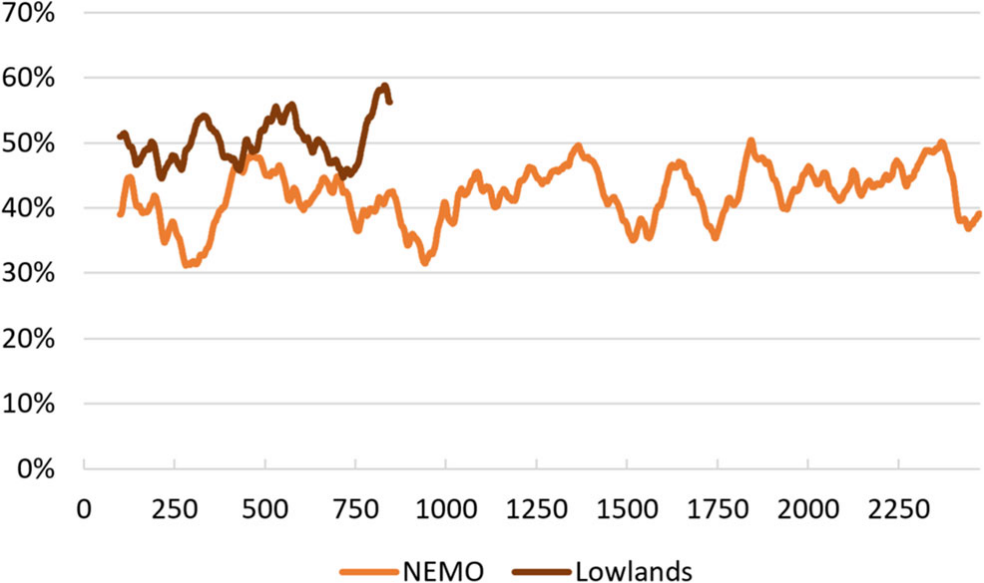
Moving average of the percentage of correct guesses by participants

Similar to the automatic gesture recognition performance, it is possible to see from the log files how well participants were able to recognize gestures performed by the robot by measuring how often participants guessed a gesture correctly. Figure 8 shows the moving average, with an interval of 100 recognition attempts and exponential smoothing (*α* = .1), of participants’ guessing performance. On average, participants guessed correctly 41.9% of the time at NEMO, and 50.3% of the time at Lowlands. Chance level in this case is between 25% (first attempt at guessing, four possible answers) and 33.3% (second attempt at guessing, three possible answers).

## Description of the resulting dataset

After deploying the system at the NEMO science museum and the Lowlands music festival, the resulting data were cleaned and then published on the Open Science Foundation^2^ as supplementary materials to this paper. The dataset includes metadata describing the participants’ age, gender, and country of residence, as well as the three-dimensional gesture recordings from the Microsoft Kinect V2 and the two-dimensional gesture recordings that were extracted from videos of participants that gave permission to have them recorded. These recordings are grouped in folders, one for each of the 35 concepts. Each filename contains the participant number, and whether this was a first or second attempt at performing the gesture. We have published the data for each of the two data collection locations separately, although they can easily be combined into a larger set by merging the folders with each other as the 35 concepts were the same between locations. The first character of the participant numbers can then still be used to tell entries from the different locations apart (N = NEMO, L = Lowlands).

Also included in the dataset are log files of all the sessions, which document the interactions that occurred (e.g., which exact gestures the robot performed, and all guessing attempts by the participants and the robot), as well as Python scripts that can be used to visualize (play back) the recordings.

### Three-dimensional recordings

**Fig. 9:**
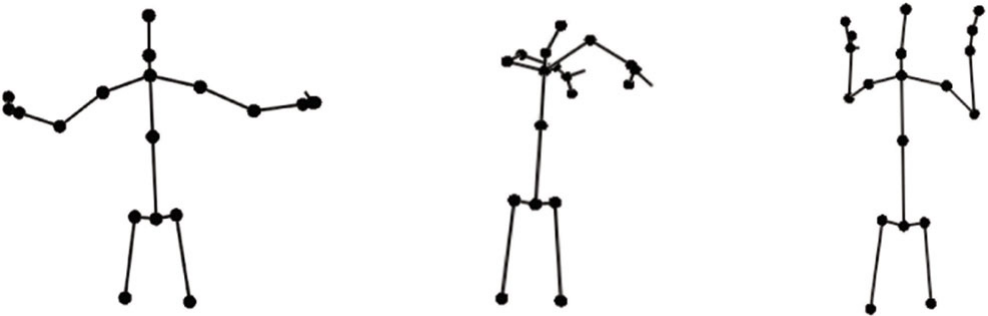
Three recordings of *bridge*, showing different ways of depicting this concept using gesture. From *left to right*, the focus is on the bridge surface, the arches, and opening of a drawbridge. The leftmost example is performed by an adult (24 years old), while the other two examples are by children (9–10 years old)

The Kinect V2 depth sensor is able to track the position of 25 different body joints (e.g., head, hips, hands, feet) at 30 frames per second. For each recording, we stored the estimated X, Y, and Z position of the 25 joints through time in a comma-separated (.csv) text file, with one line for each timestep. The Kinect uses the center of its sensor as the origin (0, 0, 0), and measures joint positions by their distance in meters from this origin. This means that the value of X increases as you move to the left of the sensor (from the perspective of the sensor, facing the participant), Y increases as you move up from the sensor, and Z increases as you move further away from the sensor. As a result, what is reported as the right shoulder was in fact the participant’s left shoulder, as seen from the Kinect sensor. In other words, these recordings will be mirrored by default when played back. Figure 9 shows a frame from three different recordings for the concept *bridge*, visualized from the comma-separated file using one of the Python scripts included with the dataset. Note that not all participants were standing far enough away from the sensor for it to be able to capture their entire body, hence the positions of their lower joints (e.g., knees and feet) could not be tracked.

In addition to the 25 joint positions, the system stored joint orientations (in X, Y, Z, W), but these appear to be redundant with the joint positions and are therefore not used in our current implementation. The estimated face orientation—an indication of where participants were looking—was also added, which has been converted into pitch, yaw, and roll values. Finally, although the sensor cannot track individual fingers, it is able to determine whether the participants’ hands are open (1), closed (0), or whether this is unknown (-1). This information was also added for each hand at every timestep, along with a confidence value indicating how sure the system was that the hand was in fact opened or closed (*Low* or *High*).

The total number of unique three-dimensional recordings is 3715. Table 2 shows how many gestures are in each subset, and how many of the recordings were first or second attempts from the same participant. Appendix A provides a more detailed overview of the number of recordings per concept for each subset.

**Table 2:** Number of three-dimensional gesture recordings per location, divided into first and second attempts

	First attempts	Second attempts	Total
NEMO	1512	1198	2710
Lowlands	561	444	1005
Total	2073	1642	3715

### Two-dimensional recordings

**Fig. 10:**
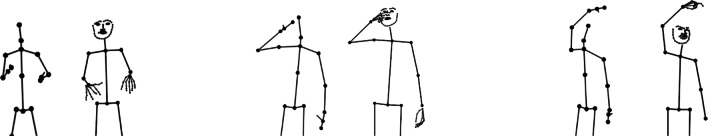
The two-dimensional and three-dimensional versions of three recordings, highlighting the advantages of having detailed hand and face motion. From *left to right*: *piano* with extended fingers, performed by a child (5 years old); *pig* by pushing the nose upward with the index finger, performed by an adult (26 years old); *stairs* with a walking motion by moving the index and middle fingers, performed by a child (10 years old)

Out of the 428 participants in our study, 367 gave permission to also have their gestures recorded on video. A Logitech C920 webcam was used, which captured the gestures at 25 frames per second. After data collection had completed, we first corrected the video recordings for the camera’s lens distortion, and then extracted motion capture data using OpenPose (Cao et al., 2017). This resulted in a similar data file to the three-dimensional Kinect recordings, including the positions of 25 body joints through time, but without depth information (the Z-coordinate). The X and Y coordinates in this case were measured in pixel locations within the video frame, which had a resolution of 1280x720 pixels, with the top left corner of the frame as the origin (0, 0). In these data the left shoulder refers to the participant’s viewpoint, so it is actually positioned further to the right than the right shoulder (which shows up on the left side of the video recording). Contrary to the three-dimensional recordings, these will therefore not be mirrored when played back. In addition to the 25 body joints OpenPose is able to track 21 keypoints on each hand (i.e., finger joints), and 70 points describing the outline and features of the face. This approach is therefore able to extract several details from video that are missing from the three-dimensional recordings, such as finger movement or facial expressions. Figure 10 shows a comparison between recordings using Kinect, and the results of running OpenPose on video recordings of the same gesture. Similar to the three-dimensional recordings, lower parts of the body such as the feet were often obscured from view and could thus not be tracked.

Because not all participants (367 out of 428) gave permission to have their gestures recorded on video, only 3269 out of the 3715 gestures could be analyzed using OpenPose. Table 3 shows how these are distributed between the two locations, and how many first and second attempts from the same participant were included. The number of two-dimensional recordings for each concept is listed in Appendix B.

**Table 3:** Number of two-dimensional gesture recordings per location, divided into first and second attempts

	First attempts	Second attempts	Total
NEMO	1284	1013	2297
Lowlands	541	431	972
Total	1825	1444	3269

### Data cleaning

Because the recording of each gesture was started by the participant, and finished after the system detected little to no hand movement for a certain amount of time, each gesture was automatically isolated and stored in the folder belonging to the right concept, in its own file, with the filename including the participant number and whether it was a first or second attempt. We have reviewed all of the recorded gestures, and identified 34 recordings from NEMO, and three from Lowlands in which no movement resembling an iconic gesture took place. These were removed from the dataset.

Although the system tried to automatically isolate the gestures, there were cases where the system prematurely detected the end of a gesture and therefore the recording was cut short. There are also examples where the system did not manage to detect the end of the gesture due to too much idle movement by the participant. Because a certain degree of noise is to be expected once interactions such as these are deployed in a naturalistic setting, we have not edited the recordings to remove these extraneous movements. The recordings might also contain participants looking at, or trying to interact with the tablet device as they double-checked the concept they were asked to perform, or if they did not realize that the recording had already started.

The tracked positions of body joints and other features are stored in a raw format, as provided by the system without any post-processing. This means that the three-dimensional recordings are currently in a different coordinate system than the matching two-dimensional versions, as described in the previous sections. Additionally, the gestures were not normalized to compensate for differences in the participants’ height, or their position relative to the Kinect and camera. Because the Kinect has a relatively wide angle of view, and because OpenPose is likely to see human-like shapes in background objects, several recordings contained data for more than one person. Recordings for which this was the case were analyzed and any measurements not related to the participant performing the gesture were removed. Finally, all data were pseudonymized, and identifiable information was removed (e.g., email addresses from the log files).

## Conclusions and discussion

In this paper, we present a large dataset of iconic gesture recordings, collected in a naturalistic setting at a science museum and a music festival. Contrary to most existing gesture elicitation procedures, in our set-up participants were free to choose how they depicted a concept by gesturing, and they were distracted from the fact that they were being recorded. With this research we aim to contribute to the fields of gesture research and human-agent interaction in two ways. First, we provide a dataset that can be used as a basis for studies into human gesturing behavior—e.g., preferred modes of representation, differences based on age or culture, and changes in gesturing strategy after miscommunication occurs—showing the degree to which variation occurs in human-performed gestures. The dataset can be used for the design of an agent’s capability to perform human-like gestures, and to recognize gestures performed by human interlocutors, taking into account this degree of variation. Second, we introduce the game of charades with a robot as a semi-structured elicitation procedure, which can be used to collect additional data in the future. To our knowledge this is the first publicly available elicitation method that employs a gameful interaction to collect gesture recordings.

This gameful elicitation method is able to bring gesture research out of the laboratory and closer to real-world settings. However, because the game restricted participants to only use their upper body, without support from speech, and because the other player was a robot that was not very good at recognizing the gestures, we imagine that the currently recorded gestures are more exaggerated (e.g., in terms of the size of the motions) than co-speech gestures used in everyday human-human conversation. It would be interesting to develop a variation of the system that is closer to the original game of charades, in which people are asked to describe an object, either using gestures or a combination of speech and gestures. In that case, the data would be less structured, because gestures no longer relate to specific cued objects but instead to object properties (e.g., ‘big’, ‘heavy’), however this would result in more broadly usable gestures. It would also be interesting to record co-speech gestures during free-form conversation with a robot, and to see if people change their gesturing behavior when their conversational partner is a robot instead of another person. The current dataset, although it contains specific gestures for 35 concepts, can be used to study various aspects of general human gesturing behavior (e.g., repair strategies, variation in preferred modes of representation). In addition, these—arguably relatively expressive—gestures are useful in domains such as foreign language education, where it is important that their meaning is especially clear, even without speech. For example, we recently used a number of gestures from this dataset in an experimental study, in which a NAO robot was used as an English language tutor for children of 4–6 years old, and the gestures were implemented to support the children’s learning process (de Wit et al., 2020).

It is important to stress that these data were collected in the field, and therefore will contain some degree of noise. There are examples where participants already started moving before the recording started, or where the system did not detect the end of the gesture properly and recorded additional movements that were no longer related to the gestures. These recordings were left as is on purpose, to give a realistic representation of the situations one could encounter when bringing this type of technology into the field, and to provide data that can be used to build solutions that can cope with these situations. As a concrete example, at one point during the experiment a participant was asked to perform a gesture for the concept *violin*, but instead showed a gesture that clearly referred to a *guitar*, another concept from our set. An additional research question that could therefore be answered using the dataset is how systems can be made intelligent enough to detect these discrepancies and handle them accordingly, for example by asking for clarification and performing the necessary relabeling autonomously.

To reduce the duration of the interaction we had to limit the number of concepts that each participant was asked to perform. Therefore, the dataset only contains recordings of five concepts per participant, instead of all 35. It is possible that the selection of concepts, and the order in which they were presented, has affected the resulting gestures. For example, both *car* and *bus* were included in the list. If participants were first presented with the cue for bus, they might only perform the act of driving, thinking that this was a unique enough description of the bus. However, if they had previously become aware that car was also included, they might have added an additional motion describing the shape of the bus, or the act of letting people board the bus, in addition to the driving motion to distinguish between the two related concepts.

The participants’ preferred strategy for depicting the concepts using gestures may have further been affected by the images that were used as prompts. For example, the image for *bridge* (shown in Fig. 2) contained a particular example with arches, which caused several participants to include an arching shape in their gesture. However, it is still unclear whether this priming effect shows for all of the included concepts. There could be concepts with a clear default mode of representation (Dargue & Sweller, 2018; Masson-Carro et al. 2017; van Nispen et al. 2014; van Nispen et al. 2017; Ortega & Özyürek 2016, 2020), which is then not affected by their representation in the images. This can be further investigated with the data we have available now, by measuring how often specific features from the images come up in the matching gestures.

There are several technical limitations to this method of data collection. The current version of the system relies on external devices—the Kinect and video camera—in order to record the gestures. We envision that in the future robots will have these features embedded, so that gesturing can become a more integral part of their abilities. This is a necessary step to make robots more inclusive by enabling them to communicate in situations where the effectiveness of spoken language is compromised, such as noisy environments or when the interlocutor has trouble understanding speech (e.g., due to being deaf or hard of hearing, or due to aphasia). In addition, the motion recording quality of the Kinect sensor is worse than that of a professional motion capture set-up. However, the portability of the Kinect, and the fact that it does not require any markers or special clothing made it more suitable to bring into a naturalistic setting such as the museum and music festival. We felt that this was also a more realistic representation of what robots of the near future would be able to do. Finally, we decided not to publish video data from the participants. Although this would have resulted in a higher level of detail, we thought that this would also increase the barrier for visitors to the museum and music festival to engage in the interaction, and might make those that did participate feel more aware of the fact that they were being recorded.

The recorded gestures were automatically mapped onto the robot, however the robot is more limited than humans in its ability to perform the gestures. As a result, it was often not clear to participants to which concept the robot-performed gestures belonged. We imagine that the performance of virtual agents or more articulate robots, both with more degrees of freedom, would be better. In future work we aim to extend the system to include support for these different agents. In addition, it might be possible to optimize the translation between the recorded gestures and the NAO robot specifically. In the aforementioned study in the field of education (de Wit et al., 2020), we applied a hybrid approach where we used recordings from the NEMO-Lowlands dataset as inspiration for the design of the gestures for a NAO robot, which were then recreated using key framing techniques (de Wit et al., 2020).

In this paper we have only provided first explorations of the dataset. There are several aspects to the gestures that can be further quantified, pertaining to the chosen modes of representation, and to the way the motions were executed (e.g., size, complexity), both within and between different concepts. We expect these aspects to be influenced by factors such as age (Jain et al., 2016; Masson-Carro et al., 2015; Sekine et al., 2018; Stites & Özçalışkan, 2017), and whether this was a first or second attempt at performing the gesture. In future work, we intend to perform a more in-depth and structured analysis of the data, in order to provide an overview of the degree of variation that exists within the set. This research can be further supported by the currently available software tools for (semi-)automatic gesture analysis (Pouw & Dixon, 2020; Trujillo et al., 2019).

In conclusion, we introduce a dataset of iconic gestures with a number of elements that set it apart from other currently available datasets: it includes a large number of recordings, from a diverse group of participants (e.g., children and adults), where participants were free to choose their gesturing method, and they were asked to perform a second attempt if the robot failed to recognize their first gesture, to provide insight into possible repair strategies that people use when non-verbal miscommunication occurs. Furthermore, the gestures were recorded by means of a semi-structured, gameful elicitation procedure. As a result, this dataset can be used for research into human gesturing behavior, and as input for various automated gesture analysis, recognition, and production algorithms. Finally, we have made the elicitation method publicly available, so that other researchers can extend the dataset in a consistent, structured manner.

## Open Practices Statement

The data and materials for all experiments are available at https://osf.io/r59hj/ and at https://github.com/l2tor/NEMO-Lowlands-charades. The experiment was not preregistered.

## Appendix A: Overview of three-dimensional recordings

**Table 4:** Number of three-dimensional recordings per concept

Concept	NEMO	Lowlands	Total
Airplane	74	25	99
Bed	80	32	112
Bird	72	27	99
Boat	74	29	103
Book	74	28	102
Bridge	78	32	110
Bus	76	30	106
Car	77	27	104
Castle	75	32	107
Chair	78	32	110
Comb	78	27	105
Cow	76	26	102
Crocodile	81	29	110
Cup	76	30	106
Drum set	75	34	109
Fish	83	25	108
Guitar	76	25	101
Helicopter	75	25	100
Horse	78	30	108
Lamp	71	26	97
Motorcycle	81	30	111
Pencil	85	31	116
Piano	78	29	107
Pig	75	29	104
Scissors	78	29	107
Spoon	80	29	109
Stairs	84	29	113
Table	79	31	110
Toothbrush	75	28	103
Tortoise	77	29	106
Train	74	25	99
Triangle	81	33	114
Trumpet	76	28	104
Violin	78	27	105
Xylophone	82	27	109
Total	2710	1005	3715

## Appendix B: Overview of two-dimensional recordings

**Table 5:** Number of two-dimensional recordings per concept

Concept	NEMO	Lowlands	Total
Airplane	57	27	84
Bed	61	32	93
Bird	68	25	93
Boat	67	29	96
Book	62	30	92
Bridge	73	32	105
Bus	65	30	95
Car	74	27	101
Castle	66	31	97
Chair	63	30	93
Comb	59	27	86
Cow	65	20	85
Crocodile	75	29	104
Cup	65	27	92
Drum set	63	32	95
Fish	72	25	97
Guitar	72	21	93
Helicopter	60	23	83
Horse	70	30	100
Lamp	58	24	82
Motorcycle	69	28	97
Pencil	76	31	107
Piano	60	29	89
Pig	68	29	97
Scissors	58	27	85
Spoon	66	29	95
Stairs	77	26	103
Table	60	28	88
Toothbrush	65	28	93
Tortoise	65	29	94
Train	63	25	88
Triangle	71	33	104
Trumpet	58	28	86
Violin	62	26	88
Xylophone	64	25	89
Total	2297	972	3269

## Footnotes

1 https://github.com/l2tor/NEMO-Lowlands-charades

2 https://osf.io/r59hj/
